# State of Art of Transcatheter Aortic Valve Implantation for the Treatment of Aortic Stenosis in Low-Risk Patients: A Perspective on Brazilian Contributions and Implementation Challenges

**DOI:** 10.21470/1678-9741-2026-0079

**Published:** 2026-11-01

**Authors:** Eduardo Keller Saadi, Rodrigo Petersen Saadi, Ana Paula Tagliari

**Affiliations:** 1 Cardiovascular Surgery Department, Hospital Mãe de Deus, Porto Alegre, Rio Grande do Sul, Brazil; 2 Department of Cardiovascular Surgery, Universidade Federal do Rio Grande do Sul (UFRGS), Porto Alegre, Rio Grande do Sul, Brazil; 3 Department of Morphological Sciences, Universidade Federal do Rio Grande do Sul (UFRGS), Porto Alegre, Rio Grande do Sul, Brazil; 4 Department of Cardiovascular Surgery, Universidade Federal de Ciências da Saúde de Porto Alegre (UFCSPA), Porto Alegre, Rio Grande do Sul, Brazil

**Keywords:** Aortic Stenosis, Transcatheter Aortic Valve Implantation, Transcatheter Aortic Valve Replacement, Brazilian Cardiovascular Surgery.

## Abstract

Transcatheter aortic valve implantation (TAVI) has profoundly changed the management of severe aortic stenosis (AS), evolving from a treatment reserved for inoperable and high-risk patients to an established therapeutic option across a wide surgical risk spectrum. Although the expansion of TAVI indications has been largely supported by randomized clinical trials conducted in high-income countries, real-world experience and technological contributions from distinct healthcare systems are increasingly relevant. Brazil has played a significant role in the early adoption, structured implementation, and clinical evaluation of TAVI in Latin America. Since the first procedures performed in 2008, Brazilian centers have developed Heart Team models, contributed to national and regional registries, and generated real-world evidence demonstrating procedural safety and effectiveness comparable to international benchmarks, despite the presence of resource limitations and a higher prevalence of complex clinical and anatomical scenarios. The incorporation of TAVI into the Brazilian public health system in 2022 marked a major milestone in expanding access to minimally invasive therapy for patients with severe AS. In parallel, Brazilian scientific societies have adapted international guideline recommendations to local practice, supporting evidence-based patient selection across different surgical risk categories. Notably, Brazil has also emerged as a developer of locally engineered transcatheter valve technologies, highlighting its transition from a technology consumer to an active contributor in the field of structural heart disease. This review summarizes the historical evolution, national experience, clinical outcomes, guideline integration, technological innovation, and health economic considerations of TAVI in Brazil.

## INTRODUCTION

**Figure:**
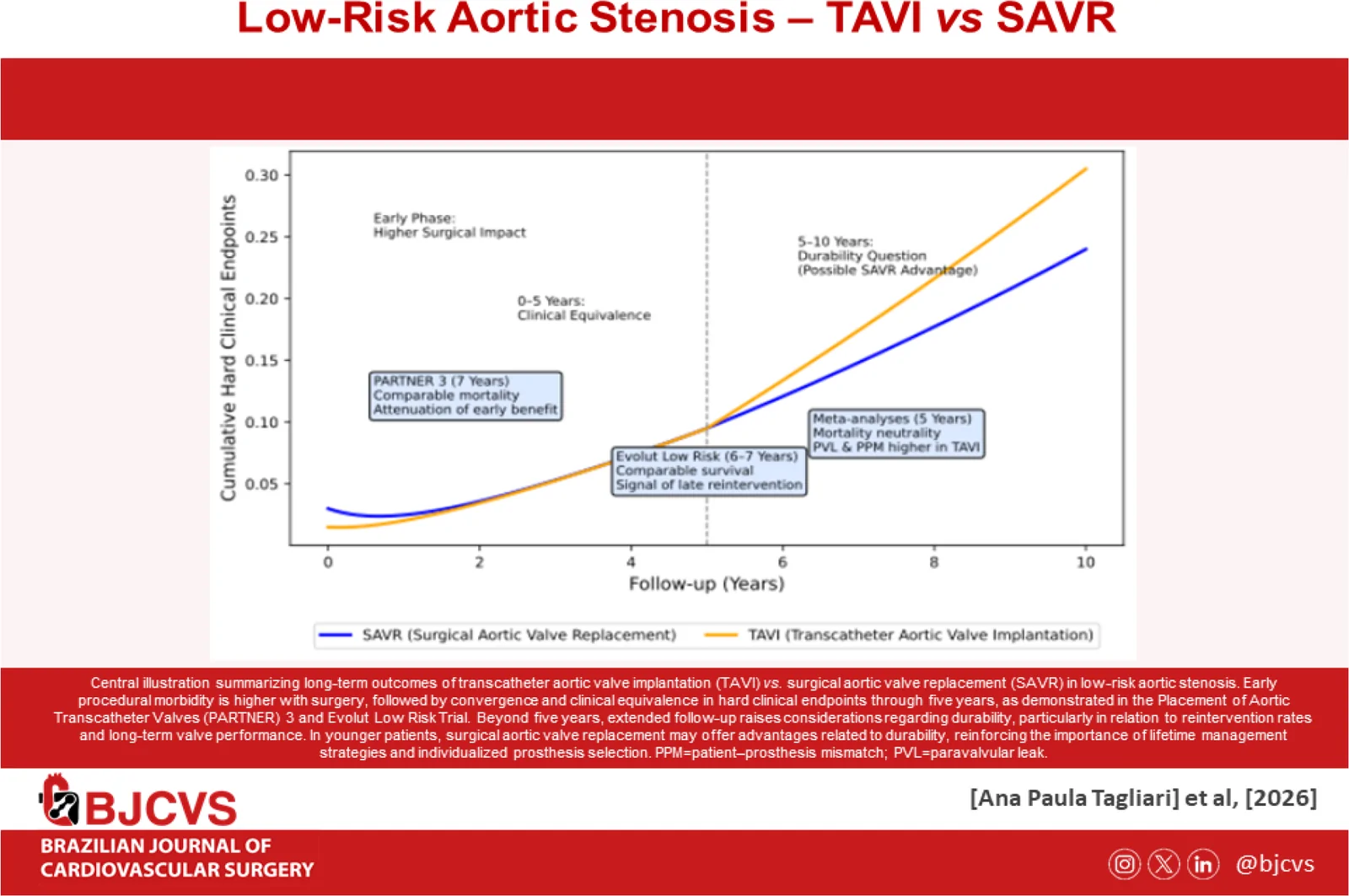

**Table:** 

Abbreviations, Acronyms & Symbols				
AS	= Aortic stenosis		SAVR	= Surgical aortic valve replacement
ESC	= European Society of Cardiology		SBCCV	= Sociedade Brasileira de Cirurgia Cardiovascular
EuroSCORE	= European System for Cardiac Operative Risk Evaluation		STS	= Society of Thoracic Surgeons
ICER	= Incremental cost-effectiveness ratio		SUS	= Sistema Único de Saúde
NOTION	= Nordic Aortic Valve Intervention Trial		TAVI	= Transcatheter aortic valve implantation
PARTNER	= Placement of Aortic Transcatheter Valves		THV	= Transcatheter heart valve
PPM PVL	= Patient-prosthesis mismatch = Paravalvular leak		WRITTEN LATAM	Recent Developments and Current Status of Transcatheter Aortic Valve Replacement Practice in Latin America

Aortic stenosis (AS) has historically been treated with surgical aortic valve replacement (SAVR), which for decades represented the standard of care. Over the last two decades, however, the advent of transcatheter aortic valve implantation (TAVI) has profoundly transformed the therapeutic landscape. Initially developed for patients deemed inoperable or at prohibitive surgical risk, TAVI has progressively expanded to intermediateand low-risk populations, supported by a growing body of high-quality evidence. Pivotal trials such as Placement of Aortic Transcatheter Valves (PARTNER) 3, Evolut Low Risk, Nordic Aortic Valve Intervention Trial (NOTION) 10-year follow-up, NOTION-2, and the recent LANDMARK trial have consistently demonstrated the non-inferiority of TAVI compared with SAVR in carefully selected patient cohorts, firmly establishing TAVI as a cornerstone of contemporary AS management across the surgical risk spectrum^[1-5]^.

Despite being classified as a developing country and often underrepresented in large multinational randomized trials, Brazil has made substantial and original contributions to the management of AS. Brazilian cardiovascular centers were among the earliest in Latin America to adopt TAVI in a structured manner, rapidly incorporating the technology into clinical practice, generating real-world evidence, and participating in the development of national registries. Furthermore, Brazil stands out globally for its role in the development of locally engineered transcatheter heart valves (THV), contributing to technological innovation, cost-effectiveness discussions, and broader access to advanced therapies in emerging healthcare systems. These contributions are particularly relevant for understanding TAVI outcomes in heterogeneous populations, including patients with advanced disease stage at presentation, complex anatomical characteristics, and treatment within resource-limited environments^[6]^.

In this context, the present article aims to provide a comprehensive and critical overview of the Brazilian contributions to the management of AS in the TAVI era. Specifically, we seek to summarize the historical evolution of transcatheter therapies in Brazil, highlight national scientific production and real-world outcomes, discuss the development of locally engineered THV devices, and analyze health-economic and policy aspects related to access within both the private and public health systems. By integrating international evidence with Brazilian data, this review intends to contextualize Brazil’s role in the global advancement of TAVI and to identify future challenges and opportunities for optimization.

### Brazilian Contributions to Aortic Stenosis Management

Brazil has a long-standing tradition of excellence in cardiovascular surgery that has substantially contributed to the contemporary management of AS and complex aortic pathologies.

One of the most influential international contributions by a Brazilian-born surgeon is that of Dr. Tirone E. David, developer of the valve-sparing aortic root replacement (David procedure). This operation revolutionized the surgical management of aortic root disease by demonstrating that preservation of the native aortic valve can provide excellent long-term durability, reduced valve-related complications, and avoidance of lifelong anticoagulation in selected patients. Although not primarily designed for degenerative calcific AS, the David procedure profoundly influenced modern concepts of valve preservation and complex aortic surgery, shaping surgical strategies for combined aortic root and valve pathology worldwide^[7]^.

Another pivotal Brazilian figure was Dr. Domingo Marcolino Braile, whose contributions extended beyond clinical surgery into biomedical engineering and health technology innovation. Through the founding of Braile Biomédica, locally developed cardiopulmonary bypass systems, prosthetic valves, and more recently a fully Brazilian-designed TAVI prosthesis have expanded access to advanced cardiovascular therapies. This technological independence represents a strategic contribution to both national healthcare sustainability and global structural heart innovation^[8]^.

The Brazilian cardiovascular surgical tradition was further strengthened by leaders such as Dr. Adib Domingos Jatene, whose influence in complex cardiac surgery, education, and health policy helped establish the institutional environment in which innovation in valve therapy could thrive. Similarly, Dr. José Honório de Almeida Palma da Fonseca played a significant role in surgical training, refinement of operative techniques, and consolidation of high-quality cardiovascular care in Brazil.

Beyond valve implantation, Brazilian groups have contributed to frontier hybrid and endovascular innovations. A notable example is the first in-human Endo-Bentall procedure, reported by Gaia et al. in the Journal of the American College of Cardiology: Case Reports. This pioneering case described a customized endovascular device combining a TAVI with an ascending aortic stent graft for simultaneous treatment of the aortic valve and ascending aorta in an inoperable patient. This work highlights Brazil’s role not only in adopting, but also in conceptualizing and executing novel structural heart solutions for complex aortic disease^[9]^.

Collectively, Brazil’s contributions to AS management encompass surgical excellence, valve-preserving philosophy, national device innovation, large-scale clinical implementation of TAVI, and pioneering hybrid interventions. These achievements illustrate how Brazilian cardiovascular surgery has influenced both local patient care and global paradigms in valvular and aortic disease management.

### Brazilian Contributions to the Development of Endovascular Aortic and Transcatheter Interventions

In the late 1990s, Dr. Enio Buffolo and Dr. José Honório de Almeida Palma da Fonseca pioneered the endovascular treatment of thoracic aortic diseases in Brazil, introducing self-expanding stent-grafts and hybrid strategies at a time when these approaches were still in their infancy worldwide. Their landmark publication in Annals of Thoracic Surgery documented the revolutionary endovascular management of descending aortic aneurysms and dissections, positioning Brazil among the early contributors to the global development of thoracic endovascular techniques^[10]^.

Subsequently, a multicenter Brazilian experience with thoracic endovascular aortic repair, involving 255 patients followed over 112 months, demonstrated procedural feasibility and provided actuarial survival analyses that anticipated contemporary discussions on long-term durability and late outcomes^[11]^.

Professor Eduardo Keller Saadi had been performing endovascular aortic procedures since 1999 and, in 2008, published one of the earliest Brazilian critical analyses addressing the feasibility and durability of TAVI. At a time when TAVI remained an emerging technology, this work reflected a forward-looking and analytically rigorous perspective^[12]^. In 2010, Saadi et al. described the axillary artery as an alternative access route for thoracic endovascular treatment, preceding the broader international adoption of transaxillary access strategies that would later become increasingly relevant in structural heart interventions^[13]^.

Beyond procedural innovation, the Brazilian Society of Cardiovascular Surgery (Sociedade Brasileira de Cirurgia Cardiovascular [SBCCV]) played a central role in reinforcing structured endovascular training and the importance of systematic long-term follow-up, thereby contributing not only to technical advancement but also to the consolidation of quality and safety standards in national practice^[14]^.

### Early Adoption of and National Experience with Transcatheter Aortic Valve Implantation

Brazil was among the first countries in Latin America to adopt TAVI shortly after its introduction in Europe. As early as 2008, reference centers, particularly in São Paulo, Rio de Janeiro, Curitiba, and Porto Alegre, started to incorporate TAVI into their clinical practice. This early adoption facilitated the development of structured Heart Team programs, accelerated procedural learning curves, and enabled the transition from compassionate use to broader clinical indications^[6]^.

A major milestone in national cardiovascular care occurred in 2022, with the incorporation of TAVI into the Brazilian Public Health System (Sistema Único de Saúde [SUS]) for the treatment of patients with severe symptomatic aortic stenosis, expanding access to this minimally invasive therapy nationwide. Brazilian professional societies - including the Brazilian Society of Cardiology (Sociedade Brasileira de Cardiologia), the Brazilian Society of Hemodynamics and Interventional Cardiology (Sociedade Brasileira de Hemodinâmica e Cardiologia Intervencionista), and the SBCCV - have played a central role in this process by contributing to the adaptation of international recommendations to local clinical realities.

Another important aspect is the Brazilian contribution to robust national registries, which have generated valuable epidemiological and outcomes data. In this context, the Brazilian Registry of Adult Patients Undergoing Cardiovascular Surgery (or BYPASS) Registry is a nationwide, prospective, longitudinal database encompassing a broad spectrum of adult cardiovascular surgical procedures. In a dedicated analysis published in 2020, including 920 patients undergoing valve surgery, the registry showed that isolated aortic valve replacement accounted for 34% of procedures, making it the most frequently performed valve operation in the country. Rheumatic heart disease remained the predominant etiology, reflecting the epidemiological profile of a middle-income country. Procedures funded by the public health system accounted for 80.8% of cases, and the overall in-hospital mortality was 7.3%, highlighting both the significant disease burden and the outcomes achievable within a resource-constrained environment^[15]^.

Specific real-world evidence on TAVI has been provided by the Registro Brasileiro de Implante de Bioprótese Aórtica por Cateter e Novas Tecnologias (or RIBAC-NT). This nationwide registry has prospectively collected data since 2008 from 266 centers across 20 Brazilian states, representing one of the largest TAVI databases in Latin America. In a comprehensive 14-year analysis covering procedures performed between 2008 and 2022, the registry demonstrated that TAVI was performed in 3,793 patients. After exclusion of cases with incomplete data, 2,588 patients were included in the final analysis (51.2% women; median age 82 years [interquartile range 78 - 86]). In-hospital mortality was 8.2%, major vascular complications occurred in 6.0%, and acute kidney injury was reported in 8.8% of cases, with no statistically significant sex-related differences in outcomes. These results demonstrate the feasibility, safety, and reproducibility of TAVI in our local reality with outcomes comparable to those reported in international real-world registries^[16]^.

Regional practice patterns and temporal trends have been further explored in the Recent Developments and Current Status of Transcatheter Aortic Valve Replacement Practice in Latin America (WRITTEN LATAM) study, a multinational survey designed to compare TAVI practice in Latin America with that of the rest of the world and to assess its evolution between 2015 and 2020. The study included responses from 250 centers in 2015 (29 from Latin America and 221 from other regions) and 46 Latin American centers in 2020, encompassing a total of 73,707 TAVI procedures. Although Latin American centers performed significantly fewer procedures than their counterparts in high-income countries, important qualitative improvements were observed over time. These improvements included a substantial increase in the adoption of TAVI minimalist approaches, reduced duration of telemetry and temporary pacing, and lower use of dual antiplatelet therapy, as reported in the WRITTEN LATAM 2020 cohort. Collectively, these findings indicate a rapid convergence of Latin American and Brazilian practice toward contemporary international standards despite persistent volume disparities^[6]^.

The Brazilian experience is particularly relevant due to unique population characteristics, including a higher prevalence of rheumatic heart disease, advanced age at presentation, delayed referral, and a high burden of comorbidities. This accumulated experience positions Brazil as a valuable contributor to global knowledge on TAVI implementation in complex, real-world settings.

### Technological Innovation: Brazil as a Developer

Brazil stands out as one of the few countries outside Europe, the United States of America, and China to develop locally engineered transcatheter aortic valves, transitioning from a technology consumer to an active developer.

The most prominent example is the Inovare® TAVI prosthesis, a balloon-expandable valve developed by Braile Biomédica. In the prospective multicenter experience published in EuroIntervention (n = 90 high-risk or inoperable patients, mean logistic European System for Cardiac Operative Risk Evaluation [EuroSCORE] 39.3%), implantation of the Inovare® valve via a transapical approach was feasible in 96.6% of cases, with only one case of operative mortality, a low conversion rate to open surgery (3.3%), and acceptable procedural outcomes in a high-risk population. The median fluoroscopy time was 11.5 ± 4.9 minutes, and the median contrast use was 29.0 ± 30.1 mL. Thirty-day all-cause mortality was 13.3%, with sustained improvements in functional class and significant reductions in both peak and mean transvalvular gradients post-implantation. Long-term Kaplan-Meier survival at 48 months was consistent with meaningful clinical benefit, emphasizing the capability of a Brazilian-designed TAVI prosthesis to achieve durable haemodynamic and clinical results^[17]^.

Building on this foundation, Inovare® SafeSync® THV represents the next generation of Brazilian transcatheter technology. Designed with a cobalt-chromium frame, external polyester sealing skirt, and radiopaque tantalum markers for precise commissural alignment, SafeSync® aims to enhance procedural accuracy and reduce paravalvular leak. It is available in multiple diameters (20 - 30 mm), enabling tailored annular coverage across a broad range of anatomies. The B-Delivery System, a deflectable transfemoral delivery platform with a low 14 Fr profile, facilitates smooth navigation through challenging vascular anatomies and optimizes positioning during deployment. The integrated features such as hydrophilic coatings, atraumatic nose cone, and hemostatic components are engineered to improve deliverability, minimize vascular trauma, and support safer percutaneous access (Figure 1).

**Fig. 1:**
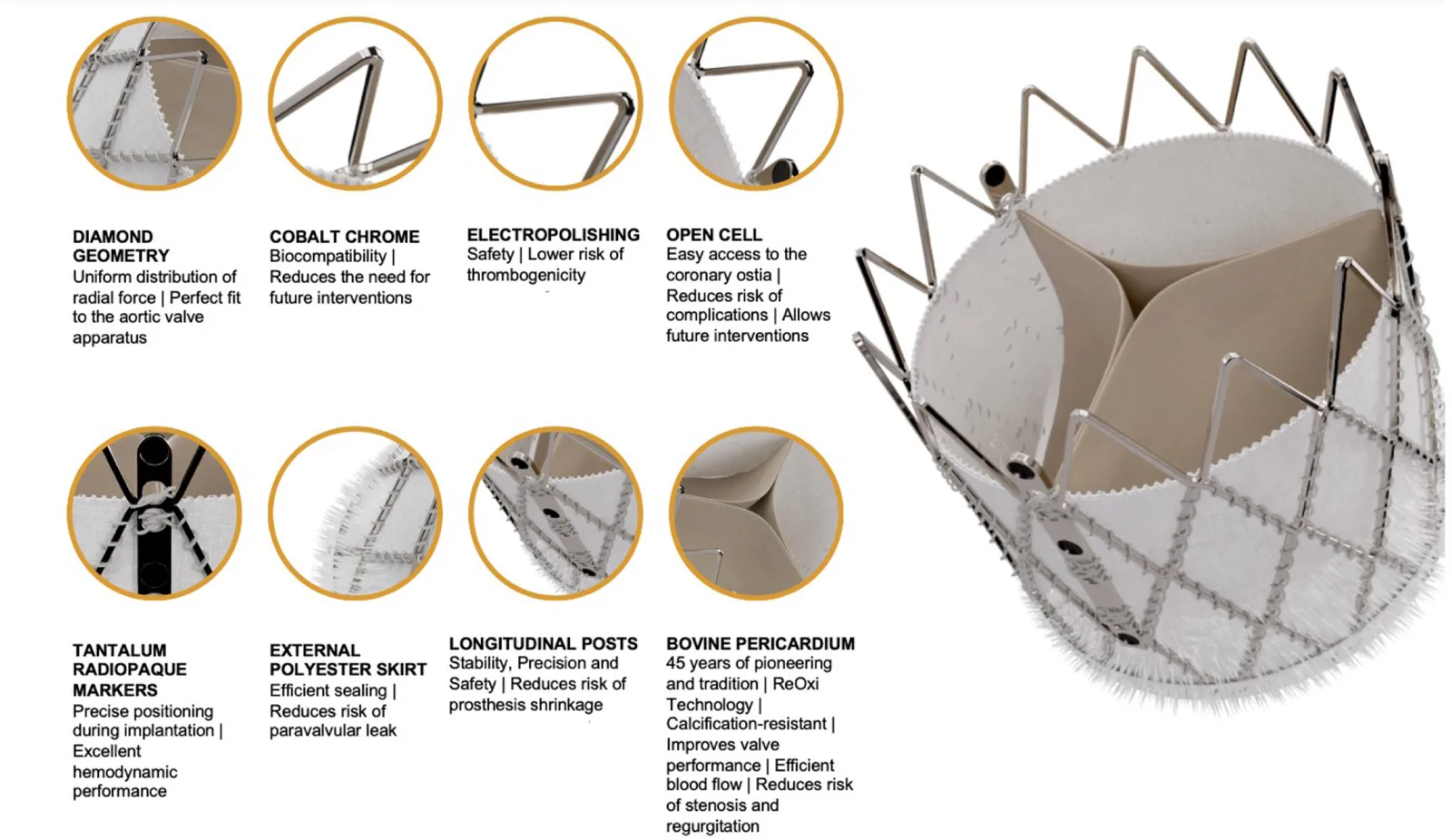
Main features of the Braile Inovare® SafeSync® transcatheter heart valve.

The development of Inovare® and its SafeSync® iteration not only underscores Brazil’s capacity for structural heart device innovation but also highlights the country’s contribution to cost-effective, anatomically adaptable TAVI solutions for emerging healthcare environments.

### The Heart Team Model and the Brazilian Heart Valve Disease Guidelines

The expansion of TAVI into lower-risk populations required not only a robust, multidisciplinary decision-making structure but also current, evidence-based clinical guidelines that support appropriate patient selection. Brazil has been a leader in implementing the Heart Team model, integrating interventional cardiology, cardiac surgery, advanced imaging, and anesthesiology into shared decision pathways that emphasize individualized treatment planning and outcomes optimization.

This integrated approach is particularly essential in discussions of lifetime management strategies, balancing the use of TAVI *vs.* SAVR in patients with low surgical risk, younger age, or complex anatomical features. Brazilian centers leverage their strong surgical tradition to ensure comprehensive involvement of cardiac surgeons in TAVI programs, including access strategy, contingency planning, and complex bail-out procedures.

The Update of the Brazilian Guidelines for Valvular Heart Disease - 2020 provided a national framework aligned with contemporary international evidence while incorporating local clinical considerations. These updated guidelines emphasize that intervention decisions for valvular heart disease, including AS, should be based on a precise anatomical and functional diagnosis, together with a comprehensive clinical evaluation and Heart Team assessment. Importantly, they recognize the role of TAVI across a spectrum of surgical risk. According to these guidelines, TAVI is recommended with the highest class of evidence (I A) for patients who are inoperable, at prohibitive surgical risk, or frail, as well as for those at high surgical risk. TAVI is also considered appropriate in intermediateand selected low-risk patients after Heart Team evaluation, particularly in individuals > 70 years of age, although the guidelines advise caution and highlight the need for durability data in younger patients^[18]^. These recommendations reflect an alignment with the current European and American guidelines while incorporating contemporary randomized trial evidence into the Brazilian context^[19-21]^.

### Health Economics and Population Access

The expansion of TAVI into intermediateand low-risk AS populations requires careful consideration of health economics, particularly in emerging economies with constrained public healthcare budgets. In Brazil, multiple studies have addressed not only clinical outcomes but also cost-effectiveness, budget impact, and system-level implications of incorporating TAVI into both the supplemental/private healthcare sector and the SUS. These contributions provide a structured framework for evaluating the sustainability of expanding TAVI indications beyond high-risk and inoperable patients.

One of the earliest and most influential Brazilian economic evaluations was published by Queiroga et al. in 2013, focusing on inoperable patients with severe AS treated within the Brazilian private healthcare system. Using a Markov model, the authors compared TAVI with standard medical therapy over fiveand 10-year horizons. In the base-case scenario, TAVI cost was estimated at R$65.000,00 per procedure. At five years, TAVI yielded higher survival (2.50 *vs.* 1.53 life-years) and resulted in an incremental cost-effectiveness ratio (ICER) of R$72.520,65 per life-year gained. When the time horizon was extended to 10 years, cumulative survival benefit increased, and the ICER decreased substantially to R$41.653,01 per life-year gained. Sensitivity analyses demonstrated that device cost and long-term survival were the primary drivers of cost-effectiveness. The study concluded that although TAVI was associated with higher upfront costs, its effectiveness and improved ICER over longer horizons supported its economic plausibility in carefully selected populations^[22]^.

More contemporary real-world data have evaluated TAVI exclusively within the SUS. Machado et al. reported a prospective observational study conducted at a public tertiary referral center in southern Brazil, including all consecutive SUS patients undergoing TAVI between July 2018 and October 2024. A total of 125 patients were analyzed (mean age 80 ± 10 years; 49.6% male; mean Society of Thoracic Surgeons [STS] score 4.6 ± 3.6%), reflecting an elderly population with predominantly intermediate surgical risk. Device success, defined according to Valve Academic Research Consortium 2 criteria, was achieved in 95.2% of cases. In-hospital mortality was low (1.6%), and major complications included new permanent pacemaker implantation in 12.8% of patients. Importantly, no significant differences in procedural success, mortality, or complication rates were observed between the early (2018 - 2022) and later (2023 - 2024) cohorts, suggesting a stable learning curve and reproducible outcomes within the SUS. One-year follow-up demonstrated outcomes comparable to those reported in international registries, reinforcing the feasibility and safety of TAVI in the Brazilian public healthcare setting^[23]^.

Complementary economic and clinical data comparing TAVI with SAVR within the SUS were provided by Saad et al. in a retrospective single-center analysis covering the period from January 2018 to December 2022. The study included 320 patients (139 TAVI and 181 SAVR). Patients undergoing TAVI were significantly older (77.9 *vs.* 64.9 years, *P* < 0.001) and had higher operative risk (STS score 3.62% *vs.* 1.64%, *P* < 0.001). Despite this risk profile, in-hospital mortality (2.9% *vs.* 5.0%) and stroke rates (1.4% *vs.* 3.3%) were numerically lower in the TAVI group, although not statistically significant. After multivariable adjustment, TAVI showed a trend toward superiority in a composite endpoint of death, stroke, and rehospitalization (5.8% *vs.* 12.0%; odds ratio 2.68; 95% confidence interval 1.04 - 7.65; *P* = 0.05). From an economic standpoint, TAVI was associated with a markedly shorter hospital stay (median 2.0 *vs.* 8.0 days, *P* < 0.001), but substantially higher total direct costs (median R$55.750,90 *vs.* R$22.518,10; *P* < 0.001), largely driven by prosthesis costs. The authors emphasized that reductions in device pricing, combined with shorter hospitalizations and potential long-term outcome benefits, could progressively improve the cost-effectiveness profile of TAVI within the SUS^[24]^. Saadi et al. reinforced this data showing that despite the higher cost associated with TAVI procedure, primarily driven by the prosthetic valve cost, it is associated with a shorter post-procedural length of stay and lower absolute one-year mortality rates (3.1% *vs.* 6.1%), although this difference did not reach statistical significance (*P* = 0.60)^[25]^.

### Implementation Challenges and Policy Aspects in Public and Private Healthcare Systems

The implementation of TAVI in Brazil has been shaped by significant structural, economic, and policy-related challenges that differ substantially between the private healthcare sector and the public health system. In the supplemental/private system, access to TAVI was facilitated by greater financial flexibility and faster incorporation of new technologies, allowing high-volume centers to develop expertise, establish Heart Team structures, and generate early national data. However, even in this setting, disparities in coverage policies, variability among insurance providers, and high device costs limited uniform access, particularly for intermediateand low-risk patients.

Within the Brazilian supplemental healthcare system, access to TAVI is formally regulated by the *Rol de Procedimentos e Eventos em Saúde* of the Agência Nacional de Saúde Suplementar (or ANS). According to the current Guidelines for Use, TAVI has mandatory coverage when all predefined criteria are fulfilled. These include symptomatic patients aged ≥ 75 years, with life expectancy greater than one year, who are deemed inoperable or at high surgical risk, defined by an STS score > 8% or a logistic EuroSCORE > 20%. In addition, the indication must be validated by a formal Heart Team, composed at a minimum of a cardiac surgeon, an interventional cardiologist, a clinical cardiologist, and an anesthesiologist, all with documented experience in TAVI procedures. This multidisciplinary team must perform a comprehensive assessment encompassing surgical risk, frailty, anatomical suitability, and comorbidities, and explicitly confirm that TAVI is more appropriate than conventional SAVR for the individual patient. While this regulatory framework ensures patient safety and appropriate resource utilization, it currently limits access to TAVI in lower-risk and younger populations, despite growing international evidence supporting its use beyond high-risk cohorts^[26]^. In these “non-regulated” scenarios, requests are typically subject to individual authorization, detailed medical justification, and, in some cases, judicialization.

In contrast, the incorporation of TAVI into the SUS occurred later and required a rigorous health technology assessment process focused on clinical effectiveness, budget impact, and cost-effectiveness. Although national and international evidence supported the clinical value of TAVI, constraints related to procedural costs, infrastructure requirements, and workforce training delayed widespread adoption. The formal inclusion of TAVI in the SUS represented a major policy milestone, yet access remains heterogeneous across regions, reflecting differences in institutional capacity, availability of trained Heart Teams, and regional funding mechanisms.

Policy discussions in Brazil increasingly emphasize the need for sustainable expansion strategies, including volume-based procurement, price negotiation, and the development of locally manufactured devices to reduce costs. Equally important are regulatory frameworks that ensure quality and safety through center accreditation, outcome monitoring, and mandatory participation in national registries. These measures aim to balance equitable access with responsible resource allocation.

As TAVI indications expand to lower-risk and younger populations, ongoing alignment between clinical guidelines, reimbursement policies, and long-term planning will be essential to ensure that technological advances translate into broad, equitable, and sustainable patient access across both private and public healthcare systems.

### Long-term Follow-up Data of Transcatheter Aortic Valve Implantation in Low-Risk Patients

In low-risk patients with severe symptomatic AS, randomized trials have demonstrated non-inferiority of TAVI compared with SAVR at shortand mid-term follow-up. Five-year outcomes from PARTNER 3 and the Evolut Low Risk Trial showed comparable mortality and stroke rates between TAVI and surgery, supporting expansion toward younger and lower-risk populations^[27,28]^.

At seven years, PARTNER 3 continued to demonstrate no significant difference between TAVI and SAVR in the composite endpoint of death, stroke, or rehospitalization (34.6% *vs.* 37.2%), with similar cumulative rates of bioprosthetic valve failure (6.9% *vs.* 7.5%) and reintervention (6.7% *vs.* 6.0%). Mean transvalvular gradients remained stable, and advanced hemodynamic deterioration was infrequent, supporting sustained valve performance. However, attenuation of the early procedural advantage of TAVI beyond the first year highlights the importance of continued long-term surveillance^[29]^.

Longer-term data from self-expanding platforms further refine this discussion. In the Evolut Low Risk Trial, sixto seven-year follow-up showed similar primary composite outcomes between TAVI and SAVR, but a higher rate of reintervention in the TAVI group, mainly driven by aortic regurgitation, with a notable proportion of these patients requiring TAVI explantation. These findings suggest that late reintervention curves may diverge beyond five years, particularly in patients who underwent aggressive post-dilatation or those with residual hemodynamic compromise. Valve platform characteristics, implantation technique, and anatomical selection therefore play a critical role in long-term durability^[30]^.

Interpretation of these findings requires caution since reintervention is an imperfect surrogate for structural valve deterioration, and factors such as valve thrombosis and subclinical leaflet abnormalities may influence long-term outcomes. In addition, differences in failure phenotype - stenotic *vs.* regurgitant - may carry distinct prognostic implications, particularly in younger patients with extended life expectancy.

Other important considerations include conduction disturbances and paravalvular leak. Permanent pacemaker implantation remains consistently more frequent after TAVI - particularly with self-expanding systems. Although early pacemaker implantation has not translated into excess mortality at mid-term follow-up, its potential long-term impact on ventricular function, especially in younger patients with longer life expectancy, remains uncertain. Similarly, paravalvular leak, while not associated with increased mortality at seven years in the PARTNER 3 trial, may still influence long-term ventricular remodeling and overall valve performance.

A contemporary meta-analysis including all low-risk randomized trials with completed five-year follow-up confirmed no significant difference in all-cause mortality (incidence rate ratio 1.01) or in the composite of mortality and disabling stroke between TAVI and SAVR. However, higher rates of paravalvular leak and permanent pacemaker implantation were consistently observed with TAVI, whereas atrial fibrillation was more frequent after surgery^[31]^.

Finally, it is important to acknowledge that, even before the publication of the long-term results from the PARTNER 3 and Evolut Low Risk trials, the NOTION trial had already provided the first randomized evidence supporting the long-term durability of TAVI. In this landmark study, 280 low-surgical-risk patients were randomized to TAVI with a firstor second-generation self-expanding CoreValve® prosthesis (n = 145) or SAVR (n = 135). At 10 years, the composite endpoint of all-cause mortality, stroke, or myocardial infarction was identical in both groups (65.5% *vs.* 65.5%). Severe structural valve deterioration was significantly less frequent after TAVI than after SAVR (1.5% *vs.* 10.0%; *P* = 0.02), while bioprosthetic valve failure remained similar between groups (9.7% *vs.* 13.8%; *P* = 0.40). Nevertheless, these findings should be interpreted with caution. The trial enrolled a relatively small number of patients (n = 280), nearly two-thirds of participants had experienced the primary composite endpoint by 10 years, and only a limited number of surviving patients remained available for serial echocardiographic assessment of valve durability. Moreover, the study evaluated old-generation transcatheter heart valves and surgical bioprostheses implanted between 2009 and 2013, which differ substantially from contemporary devices and procedural techniques. Therefore, although the NOTION trial provided the first proof-of-concept that TAVI could achieve long-term durability comparable to surgery, the more recent PARTNER 3 and Evolut Low Risk trials offer more contemporary and generalizable evidence regarding the long-term performance of current-generation transcatheter heart valves^[32]^.

The paradigm shift in the low-risk era lies in transitioning from procedural comparison to lifetime management strategy. For a 70-year-old patient, the index intervention is unlikely to be the final procedure. Anticipated valve-in-valve strategies, preservation of coronary access, risk and complexity of potential TAVI explantation, and anatomical suitability - including bicuspid morphology, small annulus, and low coronary ostia - must be integrated into Heart Team discussions. Mid-term equivalence does not automatically translate into lifetime equivalence.

In Brazil, expansion of TAVI into lower-risk populations must consider heterogeneous access to advanced imaging and follow-up, economic sustainability across public and private systems, standardization of training and center volume requirements, and the establishment of structured long-term surveillance. Adoption should balance innovation with systematic outcome monitoring to ensure durable and equitable benefit.

## CONCLUSION

TAVI in low-risk patients demonstrates midand extended-term clinical equivalence to surgery up to seven years, with comparable mortality, stroke, and valve durability. However, attenuation of the early procedural advantage, persistent differences in conduction disturbances and paravalvular regurgitation, and the unresolved question of durability beyond the first decade underscore the need for cautious expansion. In younger patients with longer life expectancy, procedural choice should be guided not only by early safety, but by a comprehensive lifetime management strategy that anticipates future reinterventions, coronary access, and long-term ventricular performance. From a Brazilian perspective, pioneering centers and the development of robust real-world evidence through national registries position the country as an active contributor to the global TAVI landscape. An emphasis on cost-effectiveness, management of complex anatomies, and the integrated Heart Team model provides a solid foundation for the safe and sustainable expansion of TAVI in lower-risk populations.

## Data Availability

The authors declare that the data supporting the findings of this study are available within the article.

## Artificial Intelligence Usage

The authors declare that no artificial intelligence tool was used in the preparation of this article.
